# Risk of malignant neoplasms in acromegaly: a case–control study

**DOI:** 10.1007/s40618-016-0565-y

**Published:** 2016-10-21

**Authors:** K. Wolinski, A. Stangierski, K. Dyrda, K. Nowicka, M. Pelka, A. Iqbal, A. Car, M. Lazizi, N. Bednarek, A. Czarnywojtek, E. Gurgul, M. Ruchala

**Affiliations:** 1 Department of Endocrinology, Metabolism and Internal Medicine, Poznan University of Medical Sciences, 49 Przybyszewskiego Street, 60-355 Poznan, Poland; 2 Department of Pharmacology, Poznan University of Medical Sciences, Poznan, Poland

**Keywords:** Acromegaly, Thyroid cancer, Colon cancer, Breast cancer, Pituitary

## Abstract

**Purpose:**

Acromegaly is a chronic disease resulting from pathological oversecretion of growth hormone and subsequently insulin growth factor-1. Several complications of the disease have been reported, including cardiovascular diseases, respiratory disorders but also increased risk of benign and malignant neoplasms. The aim of the study was to evaluate the risk of malignant neoplasms in the patients with acromegaly in comparison with the control group.

**Patients and methods:**

Medical documentation of acromegalic patients treated in one medical center between 2005 and 2016 has been analyzed. Results were compared with sex- and age-matched group of subjects with *prolactinomas* and hormonally inactive pituitary lesions hospitalized in the same department.

**Results:**

Two hundred patients with acromegaly were included. Control group was composed of 145 patients. Any malignant neoplasm in anamnesis was present in 27 (13.5 %) patients with acromegaly and six (4.1 %) subjects from control group (*p* = 0.003). Thyroid cancer was present in 14 (7.0 %) patients with acromegaly and two (1.4 %) in control group (*p* = 0.02). Breast cancer was present in seven women (5.4 % of women) in acromegaly group but none of subjects in control group (*p* = 0.02). Colon cancer—4 (2.0 %) patients in acromegaly group and 0 in control group (*p* = 0.14).

**Conclusions:**

Malignant neoplasms are significantly more common in patients with acromegaly. Particularly, risk of thyroid cancer was increased over fivefold. Systematic screening for neoplastic diseases should be important part of follow-up in these patients. Further case–control studies are strongly indicated to evaluate which neoplasms are more common in acromegalic patients and what is the exact risk of malignancy.

## Introduction

Acromegaly is a chronic disease resulting from pathological oversecretion of growth hormone (GH) and—in consequence—insulin growth factor-1 (IGF-1) [1–4]. Several complications of the disease have been reported, including cardiovascular diseases, such as hypertrophic cardiomyopathy, heart failure, hypertension, diabetes mellitus or respiratory disorders, obstructive sleep apnea but also increased risk of benign and malignant neoplasms [5–13].

The issue of the risk of benign and malignant neoplasms in acromegalic patients remains a topic of debate. Rokkas et al. [14] published a meta-analysis of colonoscopic studies, indicating significantly increased prevalence of colon adenomas, hyperplastic polyps and colon cancers. Wolinski et al. [15] performed another meta-analysis showing an increased prevalence of nodular goiter and thyroid cancer (TC). Authors described a significantly increased risk of both disorders, and pooled OR for TCs was near eight. Published data considering the presence of other neoplasms among the population of acromegalic individuals still remain very limited [9].

The aim of the study was to evaluate retrospectively the risk of malignant neoplasms in the patients with acromegaly, basing upon medical documentation from a single center, and to further compare its prevalence with sex- and age-matched control group.

## Materials and methods

### Patients

Medical documentation of acromegalic patients treated in one medical center (Department of Endocrinology, Metabolism and Internal Diseases in Poznan) between 2005 and June 2016 has been analyzed in order to find the information about the presence of any neoplastic diseases. Results were compared with control group, comprising sex- and age-matched group of subjects with pituitary microadenomas—hormonally inactive or *prolactinomas*—hospitalized in our department between 2010 and 2015.

The study was approved by The Poznan University of Medical Sciences Ethical Committee. Written informed consent was given by all participants.

### Statistical analysis

All calculations were performed using Statistica v.12 software with medical package (from StatSoft). Fisher’s exact test was used to compare the groups; *p* value under 0.05 was considered significant.

### Screening protocol

All patients hospitalized in our department undergo thyroid ultrasonography. In the case of patients with acromegaly, abdomen ultrasonography, chest X-ray and three times fecal occult blood test are performed during every hospitalization. All patients after diagnosis are also referred to the gastroenterological outpatient clinic for the further follow-up. There is no routine screening for other malignancies—in concordance with the guidelines [16]—as increased risk of malignancy was evidenced only for these two malignancies. Data on other neoplasms come from anamnesis.

## Results

Two hundred patients with acromegaly, encompassing 129 women (64.5 %) and 71 men (35.5 %), were included. Mean age was 53.3, and standard deviation (SD) 12.2 years. Control group was composed of 145 patients with pituitary microadenomas, including 99 women (68.3 %) and 46 men, mean age was 51.9, and SD 14.8 years. Differences referring to percentage of woman and mean age were not significant (*p* > 0.05).

Mean age at the time of diagnosis of acromegaly was 47.6 years, SD 13.0, and range 17–79 years. Mean duration of acromegaly at the end of follow-up was 5.6 years, SD 7.1, and range 0–38 years.

Any malignant neoplasm in anamnesis was present in 27 (13.5 %) patients with acromegaly and six (4.1 %) subjects from control group (*p* = 0.003). OR was 3.6 with 95 % confidence interval (CI) 1.5–9.0, and risk ratio (RR) was 3.3 with 95 % CI 1.4–7.7. TC was present in 14 (7.0 %) patients with acromegaly and two (1.4 %) in control group (*p* = 0.02). Among acromegalic patients, thirteen had papillary TC (PTC) and one had follicular thyroid cancer (FTC). In control group, both patients had PTC. OR for TCs was 5.4 with 95 % CI 1.2–24.1, and RR was 5.1 with 95 % CI 1.7–22.0.

Breast cancer (BC) was present in seven women (5.4 % of women) in acromegaly group but none of subjects in control group (*p* = 0.02). OR and RR are incalculable due to lack of affected subjects in the control group; OR and RR achieved by adding a constant (0.5) to all cells in the contingency table would be 12.2 and 11.5, respectively. Colon cancer was present in four (2.0 %) patients in acromegaly group and 0 in control group (*p* = 0.14). Other malignancies in the studied group included one neuroendocrine tumor of pancreas, one squamous cell cancer of the skin, one ovarian cancer and one leiomyosarcoma of the uterine. One patient had three malignancies—breast cancer, thyroid cancer and leiomyosarcoma of the uterine. In the control group, there were one neuroendocrine tumor of the duodenum, one lung cancer and one prostate cancer.

In patients with TC, mean age at the time of diagnosis was 52.6 years, SD 10.2, median 49.5, and range 39–67 years. In two patients, TC was diagnosed before acromegaly (4 years in both subjects), in three patients both diseases were diagnosed in the same year, and in nine cases TC was diagnosed posteriorly (4, 5, 8, 10, 12, 15, 16, 19 and 20 years).

In patients with BC, mean age at the time of diagnosis was 48.7 years, SD 9.2, median 51, range 36–62 years. In four patients, BC was diagnosed before acromegaly (4, 7, 7 and 16 years), in one patient both diseases were diagnosed in the same year, and in two cases BC was diagnosed posteriorly (4 and 11 years).

Taking all 29 malignant neoplasms into account, nine of them were diagnosed before acromegaly (two TCs, four BCs, two colon cancers and one leiomyosarcoma of the uterine): In one case, surgeon performing the hemicolectomy due to the colon cancer raised the suspicion of acromegaly and referred the patient to the endocrine department, and in remaining seven cases, the malignancy was diagnosed at least four years before acromegaly. In five cases (three TCs, one BC and one ovarian cancer in the metastatic phase), malignant neoplasm was diagnosed during the comprehensive examinations just after the diagnosis of acromegaly (diagnosis or suspicion made during the hospitalization in the endocrine department). Fifteen further malignancies (nine TCs, two BCs, two colon cancers, one squamous cell cancer of the skin, one neuroendocrine tumor) were diagnosed after acromegaly.

## Discussion

According to our results, patients with acromegaly presented increased risk of neoplastic disease; the risk of development of any kind of malignant tumor was over three times higher than in the control group. It is worth noticing that in group with mean age slightly over 50 years over one in eight patients was diagnosed with malignant neoplasm. It also calls attention that in majority of patients malignant neoplasms were diagnosed in parallel or subsequently to the diagnosis of acromegaly; this result indicates the great importance of systematic and comprehensive screening for neoplastic diseases in these patients.

Among malignancies, the risk of thyroid cancer was significantly and most strongly elevated with RR exceeding five. This result is quite similar to the pooled prevalence of the neoplastic transformations in this subpopulation reported in meta-analysis concerning the issue [15]. Also the increase in prevalence of breast cancer was significant, and the difference in prevalence of colon cancer was of borderline significance. Other neoplasms occurred in single patients.

Such a high number of subjects with diagnosed thyroid malignancies can be partially explained by the fact that every patient in our department—irrespective of the initial diagnosis—undergoes thyroid ultrasonography as a routine procedure. As a consequence probably all TCs were detected and described in medical documentation, including early-stage cancers which would probably remain non-symptomatic for years. In the case of other malignancies data comes mainly from anamnesis, so the real prevalence of identifiable disorders can be underestimated. On the other hand, according to the meta-analysis published by Woliński et al. [15] the pooled prevalence of thyroid cancer in acromegalic patients calculated on the basis of studies published between 2008 and 2013 was surprisingly high—almost 6 %. The current result is in compliance with this outcome.

There was a plenty of literature concerning the issue of malignant neoplasms in patients with acromegaly [5, 8, 14, 15]. However, most studies described subjects with acromegaly without comparison to control group (e.g., [3, 17, 18]) or the results were compared with the data from local cancer registries (e.g., [19–22]). Amount of case–control studies, especially describing large groups of patients, is very limited. Wolinski et al. [15] meta-analyzed the risk of TC in patients with acromegaly indicating strongly increased prevalence of the disease. However, only five case–control studies on the topic were identified. Rokkas et al. [14] combined results of studies assessing the risk of colon cancer—only three studies meeting inclusion criteria were enrolled in the final analysis. In consequence, despite the fact that results of both meta-analyses were statistically significant, confidence intervals were very broad and the precise estimation of effect size and subsequently clinical significance of findings is not possible. To give an example, Wolinski et al. [15] reported pooled OR for TC to be slightly under eight with 95 % CI 2.8–22.0. The study evidenced an increased prevalence of TC, but in fact whether we are dealing with slight increase or vast elevation of the risk remains unclear.

In the case of other neoplasms, the amount of data is very limited. Nabarro et al. [3] described high prevalence of breast cancer, about four times higher than expected, basing upon the epidemiological data. Eleven of 123 women were affected. Barris et al. [20] found only an insignificantly increased risk in comparison with the amount expected on the basis of cancer registry, and similar results were presented by Petroff et al. [23]. In the study published by Popovic et al. [24]—the only previously published case–control study assessing the prevalence of breast cancer—the risk was insignificantly higher in acromegalic patients. Four of 133 women in the study group and one of 153 women in control group presented such malignancy. Our study discloses significantly higher prevalence of breast cancer. The issue is vital as guidelines concerning management of patients with acromegaly do not include any screening for mammary tumors [16, 25]. Secondly, as some authors question the clinical significance of the increased prevalence of thyroid cancer in patients with acromegaly and consequently of active screening for that malignancy due to relatively slow development of the disease and good prognosis [26], clinical significance of breast cancer and its early diagnosis is undoubted. Further studies on the topic are strongly desirable. There are also a few reports about the increased prevalence of renal cancer and tumors of the central nervous system [8, 20] which were not supported by our results.

In conclusion, malignant neoplasms are significantly more common in patients with acromegaly. Particularly, risk of thyroid cancer was increased over fivefold; increased risk of breast cancer remains questionable but clinically important issue. Systematic screening for neoplastic diseases should be important part of the follow-up in these patients. Further case–control studies are strongly indicated to evaluate which neoplasms are more common in acromegalic patients and to assess more precisely the exact risk of malignancy.
